# CropQuant-Air: an AI-powered system to enable phenotypic analysis of yield- and performance-related traits using wheat canopy imagery collected by low-cost drones

**DOI:** 10.3389/fpls.2023.1219983

**Published:** 2023-06-19

**Authors:** Jiawei Chen, Jie Zhou, Qing Li, Hanghang Li, Yunpeng Xia, Robert Jackson, Gang Sun, Guodong Zhou, Greg Deakin, Dong Jiang, Ji Zhou

**Affiliations:** ^1^ State Key Laboratory of Crop Genetics & Germplasm Enhancement, Academy for Advanced Interdisciplinary Studies, Nanjing Agricultural University, Nanjing, China; ^2^ College of Engineering, Nanjing Agricultural University, Nanjing, China; ^3^ Regional Technique Innovation Center for Wheat Production, Key Laboratory of Crop Physiology and Ecology in Southern China, Ministry of Agriculture, Nanjing Agricultural University, Nanjing, China; ^4^ Cambridge Crop Research, National Institute of Agricultural Botany (NIAB), Cambridge, United Kingdom

**Keywords:** wheat spike detection, drone phenotyping, key yield component, yield classification, open AI software

## Abstract

As one of the most consumed stable foods around the world, wheat plays a crucial role in ensuring global food security. The ability to quantify key yield components under complex field conditions can help breeders and researchers assess wheat’s yield performance effectively. Nevertheless, it is still challenging to conduct large-scale phenotyping to analyse canopy-level wheat spikes and relevant performance traits, in the field and in an automated manner. Here, we present CropQuant-Air, an AI-powered software system that combines state-of-the-art deep learning (DL) models and image processing algorithms to enable the detection of wheat spikes and phenotypic analysis using wheat canopy images acquired by low-cost drones. The system includes the YOLACT-Plot model for plot segmentation, an optimised YOLOv7 model for quantifying the spike number per m^2^ (SNpM^2^) trait, and performance-related trait analysis using spectral and texture features at the canopy level. Besides using our labelled dataset for model training, we also employed the Global Wheat Head Detection dataset to incorporate varietal features into the DL models, facilitating us to perform reliable yield-based analysis from hundreds of varieties selected from main wheat production regions in China. Finally, we employed the SNpM^2^ and performance traits to develop a yield classification model using the Extreme Gradient Boosting (XGBoost) ensemble and obtained significant positive correlations between the computational analysis results and manual scoring, indicating the reliability of CropQuant-Air. To ensure that our work could reach wider researchers, we created a graphical user interface for CropQuant-Air, so that non-expert users could readily use our work. We believe that our work represents valuable advances in yield-based field phenotyping and phenotypic analysis, providing useful and reliable toolkits to enable breeders, researchers, growers, and farmers to assess crop-yield performance in a cost-effective approach.

## Introduction

Yield performance is in the heart of breeding, crop research and agricultural practices (Ferrante et al., 2017). The ability of reliably classifying and predicting yield production was key for plant researchers and breeders to understand crop yield performance under complex field conditions (Jin et al., 2017). Moreover, to be able to estimate yield production during the season could facilitate growers and farmers to make reliable decisions of agronomic management such as crop rotations, fertilisation, and irrigation, so that growing conditions for crops could be optimised to facilitate more accurate and sustainable agricultural practices (Reynolds et al., 2020).

In this study, we used wheat (*Triticum aestivum*) as our model plant, a key staple food in China and many countries around the world. Global wheat consumption reached 793 million tons in 2021/22 (Nduku et al., 2023), demonstrating the great significance to ensure its supply. Nevertheless, wheat yield production could be affected by many factors in the field, ranging from environmental factors to agronomic inputs (Yang et al., 2021). Hence, it is important to equip breeders and plant researchers with suitable toolkits, so that they could assess yield performance during the reproductive phase. To characterise wheat grain yield, key components such as spike number per unit area (SNpM^2^), grain number per spike (GNpS), and thousand grain weight (TGW) were often utilised (Griffiths et al., 2015). The SNpM^2^ trait was regarded as a key indicator to evaluate yield potential (Bastos et al., 2020). Breeders, crop researchers, growers and farmers often manually scored or statistically estimated this trait during field surveillance (Marza et al., 2006). However, traditional methods to quantify SNpM^2^ in the field were not only laborious, but also prone to error (Qiu et al., 2019), leading to new approaches developed to address this challenge (Furbank et al., 2019). More importantly, due to the rapidly changing climates, breeding strategies were reformed towards the improvement of crops’ climate resilience and sustainability, requiring more effective data collection and analytic tools to accelerate the process of characterising yield components (Bevan et al., 2017).

Unmanned aerial vehicles (UAVs) based plant phenotyping has been developed rapidly in the past decade (Jang et al., 2020). Due to the decreasing costs of drones and image sensors, the improvement of flight control software, and more powerful UAV-based analytic software introduced to the research field, many research groups integrated drone phenotyping into their field-based research activities (Yang et al., 2017). In order to study yield performance, a range of image sensors such as red-green-blue (RGB) cameras, multi- and hyper-spectral devices, Light Detection and Ranging (LiDAR), and thermal and infrared sensors (Kachamba et al., 2016; Gracia-Romero et al., 2017; ten Harkel et al., 2020) were utilised in drone phenotyping to acquire plant’s morphological and spectral features, from which yield-related traits and proxies could be derived (Jiang et al., 2021). For example, AirSurf applied convolutional neural networks (CNNs) to analyse millions of lettuce heads collected by manned light aircrafts, so that marketable yield of lettuce production could be estimated (Bauer et al., 2019); multi-temporal vegetation indices derived from drone-collected multi-spectral and RGB imagery were employed to predict rice grain production, showing the drone-based phenotyping could be used to identify the optimal stage for carrying out yield prediction in rice (Zhou X. et al., 2017); deep CNNs were employed to estimate rice yield performance during ripening based on aerial imagery (Yang et al., 2019); multimodal data fusion and deep learning were integrated into the classification of yield production in soybean through drone-based field phenotyping (Maimaitijiang et al., 2017); CropQuant-3D utilised open-source 3D point clouds analysis algorithms to extract canopy-level yield-related traits (e.g. 3DCI) collected by light detection and ranging (LiDAR) or drones to identify resource use efficiency wheat varieties and their yield performance (Zhu et al., 2021); AirMeasurer combined computer vision and supervised machine learning (ML) to build dynamic phenotyping algorithms to analyse yield-related traits in rice (e.g. early establishment and heading date) based on 2D/3D aerial imagery, resulting in reliable loci identified to enable the exploration of new candidate genes (Sun et al., 2022).

The above research made valuable progresses in yield-based aerial phenotyping. Still, much research aimed to establish relationships between physiological parameters (e.g. vegetation indices and canopy structural features) with yield production, which were useful proxies but did not provide a direct yield-based measure (Gizaw et al., 2018). Due to the rapid advances in vision-based artificial intelligence (AI) and deep learning (DL), AI-powered techniques such as object detection, classification, semantic segmentation, and pattern recognition opened a new door for yield-based trait analysis (Wang et al., 2020). For example, SpikeSegNet (Misra et al., 2020) used an encoder-decoder with hourglass architecture to detect wheat spike signals for indoor experiments; DeepCount (Sadeghi-Tehran et al., 2019) combined simple linear iterative clustering and deep CNNs to identify wheat spikes in the field, indicating the feasibility of applying DL to detect spike-like objects but was limited in scale and varieties; by tilting camera angles, DL models were trained to count wheat spikes and estimate yield when spike density was low (Hasan et al., 2018); fully convolutional network (FCN) and transfer learning were employed to perform semantic segmentation of wheat spike regions using time series collected by CropQuant workstations (Zhou J. et al., 2017; Alkhudaydi et al., 2019), demonstrating the usefulness of AI-powered trait analysis of key yield components.

The above methods verified that DL-based approaches could bring unique values to the detection of key yield components such as wheat spikes under field conditions. Nevertheless, due to the complex field conditions and the large-scale nature of field trials, ground-based stationed phenotyping devices were rather limited if hundreds of plots needed to be examined. Consequently, drone-based field phenotyping was likely to bridge the gap between accuracy and scalability in yield-based studies (Yang et al., 2017). While the above ML/DL methods advanced our capability in detecting wheat spikes, the generality and scalability of them needed to be improved due to diverse wheat spike morphologies (e.g. awned, awnless, long and short spikes) and recent advances in vision-based AI research (Patrício and Rieder, 2018). In fact, besides morphological and spectral features, semantic information should also be considered in the detection of wheat spikes from the canopy (Sadeghi-Tehran et al., 2019). Hence, domain knowledge such as wheat spike developmental (e.g. key growth stages) features and plot- and organ-level morphological features should be taken into consideration when building DL models (Zhou et al., 2022), so that wheat spikes could be identified reliably under field conditions, including colour changes caused by changing natural illuminance, clustered or sparse spikes due to dissimilar growth paces, or canopy-level spike occlusion during the reproductive phase.

Our work, CropQuant-Air, presents an open and AI-powered software system that combined state-of-the-art DL techniques into the detection of wheat spikes under complex field conditions. The CropQuant-Air system first integrated the YOLACT-Plot model, a DL model based on the YOLACT++ model (Angeles Ceron et al., 2021), to enable the automated plot segmentation based on drone-collected wheat canopy image series. Within the segmented plots, an optimised YOLOv7 model (Wang et al., 2022) was trained using our labelled spikes together with the Global Wheat Head Detection (GWHD) dataset (David et al., 2020), which was employed to perform spike detection and quantify the SNpM^2^ trait. Moreover, we have included a range of image processing algorithms in CropQuant-Air to conduct canopy-level trait analysis using spectral and textural features possessed by the aerial images. To verify the CropQuant-Air system, we applied it to a field experiment studying 210 wheat varieties (two replicates; 420 plots) selected from main wheat production regions in China. Besides the analysis of the SNpM^2^ and canopy-level traits, we also developed the Extreme Gradient Boosting (XGBoost) ensemble (Chen and Guestrin, 2016) to classify yield groups using the quantified yield- and performance-related traits. After that, we performed correlation analysis between the computational analysis results and manual scoring on target traits and obtained significant positive correlations, indicating the reliability of the CropQuant-Air system in AI-powered phenotypic analysis. Finally, to ensure that our work could reach the broader plant research community, we created a graphical user interface (GUI) for CropQuant-Air, so that non-expert users could readily use our work for their yield- and performance-related trait analysis.

## Materials and methods

### Plant materials and field experiments

In order to verify trait analysis results generated by CropQuant-Air, we selected 210 winter wheat varieties cultivated for several main wheat production regions in China including Jiangsu (East China), Shandong (North China), and Henan (Central China). These lines were known for their dissimilar yield performance and different spike morphologies (Betts et al., 2014), which were suitable for building generalised DL models to incorporate dissimilar phenotypic variation into the AI-powered plot and spike detection based on canopy-level wheat images (see **Table S1** in **Supplementary Material** for the list).

**Figure 1:**
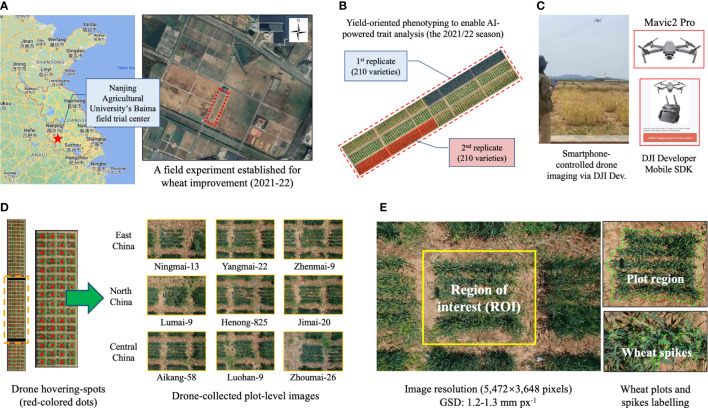
The field experiment and plot-based images acquired by low-cost drones. **(A, B)** Geo-location of the trial centre, where 210 wheat varieties were studied; the varieties were selected from three main wheat production regions in China. **(C, D)** The aerial phenotyping using a low-cost drone and wheat canopy imagery acquired from an overhead perspective, with some representative varieties listed. **(E)** The image resolution of the collected aerial images together with plot- and spike-level image annotation.

The field experiment was conducted at the Nanjing Agricultural University’s Baimai field trial center (Nanjing China; 31°36’57.8” N, 119°10’46.1” E; red coloured 5-point star, **Figure 1A**), just below 0.6 hectares (ha) in size. During the 2021/22 growing season, the 210 wheat varieties (two replications; dark blue and dark red shading areas; **Figure 1B**) were sown in 1.5 × 1.5 m plots, with 20 cm spacing, 5 rows per plot, and around 450 plants per plot. At the end of the season, we threshed and weighted the dry grains to measure grain production per plot (in kg), grain production per m^2^ (GPpM^2^), and thousand grain weight (TGW, in kg). The yield data was manually classified into three groups (i.e. high, medium, and low) according to protocols previously published (Leilah and Al-Khateeb, 2005), which were also used as groundtruthing when verifying the yield classification model.

### Drone-based phenotyping

As we focused on collecting canopy-level features using low-cost drone phenotyping, we therefore developed a smartphone-based flight control function based on the DJI developer Mobile SDK (DJI, Shenzhen, China), which could control small drones (i.e. Mavic2 Pro, equipped with an high-definition RGB camera, with a maximum image resolution of 5,472 × 3,648 pixels). After verifying the image resolution for AI-powered detection as well as the speed of drone phenotyping, we chose to fly the drone to image wheat canopy from an overhead perspective at a 4-m altitude during late flowering and early grain filling stages, so that we could accomplish the aerial imaging within 30 minutes and with AI-compatible wheat imagery (**Figure 1C**). Also, due to the aviation regulation in China, we manually flew the drone via an Android smartphone to hover at fixed spots directly above target plots and imaging was conducted using an auto-ISO mode with a fast fixed shutter speed (**Figure 1D**, left). Two series of images were generated during the phenotyping, consisted of 210 aerial images, representing the 210 winter wheat varieties’ morphological and spectral properties at the canopy level (**Figure 1D**, right). After each flight, the acquired images were transferred to a cloud server (Baidu Netdisk, Beijing, China) to enable different project partners to review and pre-process. Some testing files were uploaded to our GitHub repository for academic research and development (R&D) activities.

### The training dataset for AI-powered trait analysis

AI-powered plant phenomics research heavily relied on high-quality labelled data. To extract wheat plots (i.e. regions of interest, ROIs; **Figure 1E**, left) from acquired aerial images effectively, we first employed the Labelme toolkit (Torralba et al., 2010) to label plot outlines (480 labelled plots in total, in the COCO2017 format); then, we applied the Labelimg tool (Yu et al., 2019) to annotate wheat spikes within the ROIs (212,596 spikes in total, stored in the PASCAL VOC format). Some of the labelled plot- and spike-level datasets (**Figure 1E**, right) was also uploaded to the GitHub repository.

**Figure 2:**
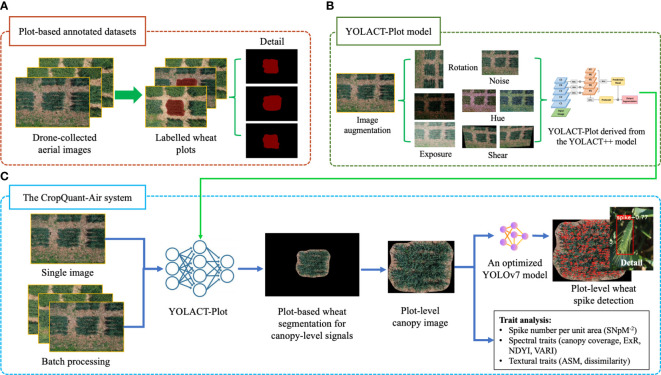
The analysis workflow of the CropQuant-Air system established for extracting plot-level phenotypic traits from drone-collected wheat canopy images. **(A)** The establishment of wheat-plot training data to identify wheat plot region using the Labelme toolkit. **(B)** Image augmentation applied to enhance the wheat-plot training dataset. **(C)** The CropQuant-Air system developed for processing drone-collected wheat images and quantifying phenotypic traits based on morphological and spectral signals.

The wheat-plot training dataset was used to enable the AI-powered plot detection, so that the central plot region in a given aerial image could be reliably identified (**Figure 2A**). To improve the wheat-plot training dataset in terms of unevenly distributed samples (i.e. dissimilar variety numbers from the three wheat production regions), we applied image augmentation techniques to enhance the dataset, including techniques such as luminance enhancement, random rotation, pretzel noise, and mosaics (**Figure 2B**; middle), resulting in a total of 1,920 annotated plots, which were divided into training (1,351 images; 70%) and testing (579 images, 30%) sets.

The wheat spike annotation was conducted by three specialists, whose labels were combined as the wheat-spike training dataset. Besides our own imagery, we utilised training datasets previously published, i.e. the GWHD dataset (collected by nine organisations, covering genotypes from Western Europe, North America, Australia and East Asia), where an open and diverse dataset of wheat spikes were labelled from RGB images for developing and benchmarking ML/DL models. In order to improve the generalisation and accuracy of our AI-powered wheat spike detection algorithm, we combined GWHD and our wheat-spike training data with image augmentation techniques to train the YOLOv7 (Wang et al., 2022) based wheat spike detection model.

### The algorithmic workflow of phenotypic analysis

We developed a three-step algorithmic workflow to incorporate computer vision and DL algorithms into the plot-based trait analysis: (1) a YOLACT-Plot segmentation algorithm (**Figure 2B**; right) was trained to enable the detection of wheat plots in aerial images; (2) then, canopy-level signals within the segmented plot were analysed by computer vision and DL-based object detection algorithms, resulting in the measurement of a range of phenotypic features, including plot-level wheat spikes, spectral (i.e. excess red vegetation index, ExR; normalised difference yellowness index, NDYI; visible atmospherically resistant index, VARI) and textural traits (i.e. canopy coverage; angular second moment, ASM; greyscale co-occurrence matrices, GLCM dissimilarity); (3) finally, plot-based trait analysis results (in CSV) and processed images (i.e. plot region segmentation and plot-level spike detection; in JPG) were produced and downloadable via the CropQuant-Air system (**Figure 2C**). When calculating spectral traits, we followed the approach that was developed for RGB-sensor-based trait analysis without radiation calibration (Svensgaard et al., 2021).

### The YOLACT-Plot segmentation model

**Figure 3:**
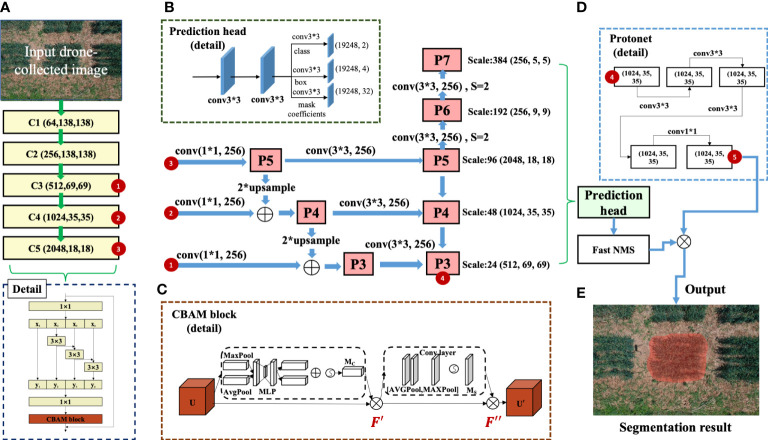
The learning architecture of the YOLACT-Plot model, which was built on the YOLACT++ model together with wheat plot training data. **(A)** The backbone architecture of the improved YOLACT-Plot model. **(B, C)** The architecture with improved Res2Net together with the prediction head as well as the CBAM block with attention mechanism. **(D, E)** The Protonet block and the plot segmentation result produced by the learning model.

The YOLACT++ model was an enhanced fully-convolutional model built for real-time instance segmentation. We adopted its learning architecture and built the YOLACT-Plot model to identify wheat plots within aerial images. The YOLACT++ network was composed of a ResNet101 backbone network to extract features from input images, generating five feature maps (i.e. from C1 to C5; **Figure 3E**). Following the standard architecture, we utilised the C3-C5 feature maps (red coloured numbered circles 1-3) as input layers of the feature pyramid, which were fused to produce five sub-feature maps (P3 to P7) at different scales (**Figure 3B**). The ResNet-101-based backbone network in the YOLACT++ employed the Bottleneck Residual structure as the fundamental module to enhance feature extraction and address the gradient vanishing problem (Wu et al., 2019). In our case, different sizes and shapes of plots needed to be detected under complex field conditions. Hence, we optimised the model by replacing the Bottleneck module with a modified Res2Net module (Gao et al., 2021), which facilitated the extraction of deeper and high-level features contained in a single layer. The Res2Net module consisted of four feature sub-graphs that had the same spatial size and channels, whose output was convolved 3 × 3 with the previous feature sub-graph as its input. Finally, the outputs of the four feature sub-graphs were combined via a 1 × 1 convolution, enabling the reuse of features to help us expand the perceptual domain to facilitate the extraction of both global and local information (**Figure 3A**; below).

Additionally, to further the enhancement of feature extraction in the YOLACT-Plot model so that invalid or irrelevant features could be suppressed, we added a Convolutional Block Attention Module (CBAM) to the Res2Net module (Woo et al., 2018). The CBAM block (**Figure 3C**) had two components: (1) channel attention and (2) spatial attention, which could generate weights of one-dimensional channel attention from the feature map as well as two-dimensional spatial attention. The channel attention component obtained the input feature map U (in H × W × C format, C represents the number of feature map channels) through global maximum pooling and then average pooling, producing two 1 × 1 × C feature maps, both of which were fed into a two-layer perceptron with shared weights. The output features were summed and activated to produce channel attention weights $M_{c}$ (Eqns. 1-3). Finally, the input feature map and the channel attention weights were multiplied to generate the channel attention feature map $F^{\prime }$ (Eqn. 4), enabling the model to focus both high- and low-level features of target plots and hence the improvement of YOLACT-Plot’s feature extraction.


(Eqn. 1) $$Z_{avg}=\text{AVG}Pool\left(\text{U}\right)$$

(Eqn. 2) $$Z_{max}=\text{MAX}Pool\left(\text{U}\right)$$

(Eqn. 3) $$M_{c}=\text{σ}\left[f_{2}\left(\text{δ}\left(f_{1}\left(Z_{avg}\right)\right)\right)+f_{2}\left(\text{δ}\left(f_{1}\left(Z_{max}\right)\right)\right)\right]$$

(Eqn. 4) $$F^{\prime }=M_{c}\times U$$

where  AvgPool  denotes the global average pooling, averaging the pixel-based intensity values of each channel; MaxPool denotes the global maximum pooling, preserving the maximum intensity value of each channel’s feature map; $f_{1}\;$ represents the fully connected layer of an input channel C for the CBAM and output channel C/16; $f_{2}\;$ represents the fully connected layer of input channel C/16 and output channel; δ signifies a rectified linear unit (ReLU) function; σ signifies the Sigmoid function.

The spatial attention component used the input feature map $F^{\prime }\;$ through the average pooling and maximum pooling of channel dimensions to obtain two H × W × 1 feature maps, which were combined and then passed through a 7 × 7 convolution layer, followed by the Sigmoid function to obtain the spatial attention weights $M_{c}$ (Eqns. 5-7) from a single channel in two dimensions. Finally, multiplying the spatial attention weights with the channel attention feature map resulted in a new feature map $F^{\prime \prime }$ , with both spatial and the channel attention features (Eqn. 8).


(Eqn. 5) $$Z_{AVG}=\text{AVGPool}\left(\text{U}\right)$$

(Eqn. 6) $$Z_{MAX}=\text{MAXPool}\left(\text{U}\right)$$

(Eqn. 7) $$M_{c}=\text{σ}\left[f_{3}\left(Z_{AVG},Z_{MAX}\right)\right]]$$

(Eqn. 8) $$F^{\prime \prime }=M_{s}\times F^{\prime }$$

where AVGPool denotes channel dimensional averaging pooling, where pixel values corresponding to each channel’s feature map are summed and averaged; MAXPool denotes maximum pooling of channel dimensions, where the maximum pixel intensity value of teach channel’s feature map are retained; $f_{3}$ represents a convolution of size 7 × 7, with an output channel of 1; σ signifies the Sigmoid function.

Building on the refined feature maps (C1-C5) and the above attention mechanism, P3-P7 sub-feature maps were fed into two parallel branches to perform plot detection: (1) the ProtoNet branch (square-dotted rectangle; **Figure 3C**, upper), which generated *k*-prototype masks with varying regional responses from the P3 feature map (red coloured numbered circle 4 in the square-dotted rectangle; **Figure 3D** upper); (2) the prediction head (square-dotted rectangle; **Figure 3B** upper left), which produced anchor frames with varied aspect ratios (i.e. 1, 1/2, 2, 1/3, 3) that employed pixel-based points of the output feature map as anchor points to detect instances, followed by an anchor-frame classification and the coefficient prediction. Both Fast Non-maximum Suppression (NMS) filters and Bounding Box Regression were applied to screen all the candidate detection boxes, resulting in instance prediction that was linearly combined with the prototype mask coefficients and hence the final mask obtained after the auto-thresholding (**Figure 3E**).

### YOLOv7 for wheat spike detection

We combined the annotated wheat spikes in GWHD and our annotated wheat spikes to train a detection model based on YOLOv7 as the baseline model. YOLOv7 was an efficient and accurate object detector that was suitable for detecting small objects in regions with dense target objects. Particularly, we chose YOLOv7 to detect canopy-level wheat spikes when they were densely clustered, occluded, or under varied nature illuminance because the YOLO-based model was suitable for recognising objects in crowded scenes in a high-throughput and high-accuracy manner (Chen et al., 2021). As a result, we chose the Standard version of YOLOv7 due to the easiness of the software deployment and reasonable computational cost. The detailed implementation of other versions of YOLOv7 (e.g. Tiny and W6) can be found via https://github.com/WongKinYiu/yolov7.

### Model training strategies

We built a workstation (Intel Core i7-8700 CPU, Nvidia 3060 GPU, and 64 GB RAM) to conduct the DL training. Both PyTorch (v1.11) framework (Paszke et al., 2019) and Python (v3.8) were utilised to implement and train the above two DL models, one for plot segmentation and one for wheat spike detection. During the training, an input images were first resized (550 × 550 pixels) and then trained with optimised parameters (e.g. batch size = 8; stochastic gradient descent (SGD) momentum = 0.9; learning rate = 0.001; epochs = 500). The loss value was used as an evaluation metric to quantify the deviation between the predicted results and the labelled data (i.e. the lower the loss value, the higher the prediction accuracy) through each iteration during training. We applied the binary cross-entropy as the loss function. When the number of iterations increased, the loss value decreased, indicating an improved performance. The loss value of the YOLACT-Plot model stabilised at approximately 0.52 after 800 iterations, whereas the optimised YOLOv7 model stabilised at around 0.22 after 1,000 iterations.

### Model evaluation

We selected average precision (AP) as the performance metric to evaluate the segmentation result. AP was calculated using Eqns. 9-11. To ensure a comprehensive evaluation of the performance of the DL models, AP50 (intersection over union, IoU = 0.5), AP75 (IoU = 0.75), and the mean Average Precision (mAP) were employed since the selection of IoU could influence the precision and recall scores.


(Eqn. 9) $$P=\frac{TP}{TP+FP}$$

(Eqn. 10) $$R=\frac{TP}{TP+FN}$$

(Eqn. 11) $$\text{AP}=\int_{0}^{1}P\left(R\right)dR$$

where *TP* denotes the number of samples where the predicted category of the model matches the true labelled category; *FP* indicates the number of samples where the predicted category does not match the true labelled category; *FN* denotes the number of samples where the predicted category is the background, but the true labelled category are other categories.

The complexity of the learning model was evaluated using the number of parameters and floating-point calculations (*FLOPs*). Since the activation functions and biases affect the calculation of *FLOPs*, different calculations were performed. To ensure consistency of our analysis, we used PyTorch’s third-party library, Thop (Jian et al., 2022), to calculate model parameters and *FLOPs*. For a single convolution operation, the model parameters and *FLOPs* were calculated using the equations below:


(Eqn. 12) $$Parameter=K^{2}\times C_{in}\times C_{out}+C_{out}$$

(Eqn. 13) $$FLOPs=C_{in}\times K^{2}\times C_{out}\times H\times W$$

where $C_{in}$ and $C_{out}$ are the number of input and output feature map channels; K denotes the size of the convolution kernel; H×W stands for the size of the output feature map.

### GUI design and software implementation

Finally, to ensure that our AI-powered phenotypic analysis system could reach the broader research community, we created a graphical user interface (GUI) for nonexperts. The CropQuant-Air GUI followed a modular architecture and implemented using the Python programming language. The cross-platform GUI (in EXE) integrated the above trained AI models and trait analysis algorithms into a stepwise software system and was developed using the Tkinter library (Shipman, 2013). To implement phenotypic analysis and ML/DL libraries, we employed open-source libraries such as SciPy (Virtanen et al., 2020) for scientific data processing, OpenCV (Bradski and Kaehler, 2008) for image analysis, Scikit-Learn (Pedregosa et al., 2011) for yield classification modelling, and the AirMeasurer libraries (Sun et al., 2022) for developing phenotypic trait analysis.

### Yield classification model and statical analysis

In order to train a ML-based model to classify yield production, we used the seven traits produced by the CropQuant-Air (i.e. SNpM^2^, NDYI, ExR, VARI, canopy coverage, ASM and GLCM dissimilarity) as input parameters and the manually measured yield production (i.e. GPpM^2^ and TGW) as targets. After testing a range of supervised ML models, we chose a stochastic gradient boosting algorithm, extreme gradient boosting (XGBoost) ensemble, as it provided the best performance compared with other supervised ML classifiers. When training the model, we fine-tuned the hyperparameters of the XGBoost model, including the number of trees, tree depth, learning rate, the number of samples, and the number of features. A grid-based search was employed to fine-tune the model to yield optimised hyper-parameters, followed by a combination of parameters with reasonable ranges of parametric values to simplify the procedure. Finally, *k*-fold cross-validation was adopted to evaluate the model performance, based on which the model with the best accuracy was selected. The above algorithmic steps and software implementation were performed using the Scikit-Learn library and saved in a separate executable Jupyter notebook, which can be downloaded from our GitHub repository.

## Results

### Datasets collected from the study

We collected two series of aerial images from the field experiment between booting and early grain filling using the low-cost drone, over 15.3 GB in total. The plot- and spike-training datasets were built using these images, including 420 annotated plots, 212,596 labelled wheat spikes, and augmented sub-images (2,940 for plots and 1,488,172 for spikes), covering the 210 varieties. During post-harvest handling, plot-based yield production, GPpM^2^ and TGW were manually quantified from the 420 plots, which were randomly divided into 70% and 30% datasets for training, testing and validation to build the yield classification model.

### Plot segmentation using the YOLACT-Plot model

**Figure 4:**
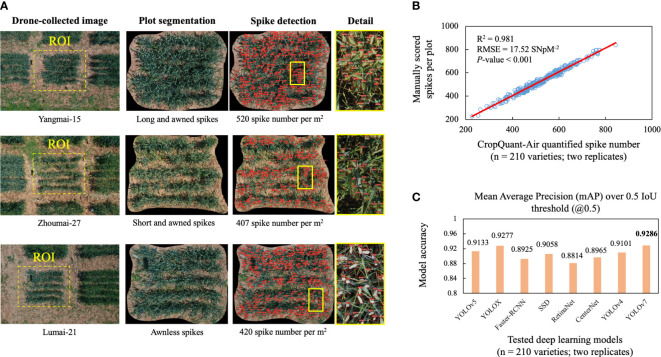
Plot- and spike-based detection using the trained YOLCAT-Plot and YOLOv7 models together with correlation analysis between AI-derived and manually scored spike number per m^2^ (SNpM^2^), followed by comparisons of eight AI models for wheat spike detection. **(A)** Plot regions detected from aerial images using YOLACT-Plot model, followed by the optimised YOLOv7 model established for detecting wheat spikes. **(B)** Correlation analysis performed between AI-derived and manually scored wheat spikes using 420 plot-based wheat canopy images, showing a significant positive correlation. **(C)** Performance comparisons of eight state-of-the-art AI-powered object detection methods, indicating that the optimised YOLOv7 model performed the best in terms of the mean average precision at intersection over union (IoU) thresholds of over 0.5 (mAP@0.5).

We applied the YOLACT-Plot model to identify the central plot in a given aerial image (**Figure 4A**; first column). To evaluate the impacts of different components or hyper-parameters on the plot detection, we conducted an ablation study (Meyes et al., 2019), which compared different components in the model and identified the essential factors for the plot detection. Three sets of experiments were accomplished (**Table 1**). The 1^st^ and 2^nd^ experiments suggested that the introduction of the Res2Net module in the backbone network improved the mAP of the prediction frame (2.35%) and mask (1.76%); whereas the 2^nd^ and 3^rd^ experiments indicated that the Res2Net-CBAM module improved the mAP of the prediction further (1.89% and 0.85%, respectively), with slightly decrease in speed (i.e. 1 frame per second, FPS).

**Table 1:** Ablation experiments conducted to identify key components essential for better detection performance in the YOLACT-Plot model.

Ablation experiment ID	Backbone network	FPS	Frame mAP%	Frame AP75%	Mask mAP%	Mask AP75%
1	ResNet-101	28.9	62.34	73.11	61.69	68.91
2	Res2Net	26.7	64.69	76.01	63.45	70.75
3	Res2Net-CBAM	27.7	66.38	77.13	64.30	72.36

With an improved backbone network, features from different channels of the same feature layer were combined for multiple times, facilitating the extraction of semantic information while leading to an increase in the size and parameters of the model. **Table 2** listed the number of parameters and the total *FLOPs* before and after the optimisation, suggesting that the YOLACT-Plot model improved in the network parameters (9.8%), model size (1.06%), and *FLOPs* (6.7%) compared with a standard YOLACT++ model.

**Table 2:** Comparison of parameters and FLOPS before and after improving the model.

Model	Parameter size/MB	Model size/MB	FLOPS/GFLOPS
YOLACT++	31.62	126	15.36
YOLACT-Plot	33.79	129	16.77

To further evaluate the YOLACT-Plot model in instance segmentation, we compared the model with several state-of-the-art instance segmentation DL algorithms such as Mask R-CNN, SOLOv2, and YOLACT++ (Zhao et al., 2021). Our wheat-plot training data was also used when training and testing these DL models. **Table 3** listed the results, indicating that the YOLACT-Plot model outperformed Mask R-CNN, SOLOv2, and YOLACT++ in the mAP by 4.15%, 5.33% and 4.1%, respectively. Hence, the feature extraction capability in the YOLACT-Plot model was clearly enhanced due to the optimisation of the learning architecture.

**Table 3:** Result comparison between DL models using the wheat-plot training dataset.

DL models	Backbone network	FPS	Mask mAP%	Mask AP75%	Mask AP50%
Mask R-CNN	ResNet-101	25.8	61.21	68.21	84.10
SOLOv2	ResNet-101	29.6	60.03	68.31	85.87
YOLACT++	ResNet-101	25.1	61.26	69.43	86.33
YOLACT-Plot	CBAM-ResNet-101	21.2	65.36	72.60	87.39

### Wheat spike detection using the optimise YOLOv7-based model

In order to evaluate the YOLOv7-based model for wheat spike detection within an identified plot, we have performed correlation analysis based on the 210 varieties possessing varied spike morphologies such as long and awned, short and awned, and awnless varieties (**Figure 4A**; second column). The detected wheat spikes, red-coloured binding boxes and confidence levels were also generated by the model (**Figure 4A**; third and fourth columns). We arranged three technicians to manually score the number of wheat spikes per plot using the same plot images segmented by the YOLCAT-Plot model. Coefficient of determination (R^2^) was computed to evaluate correlations between the CropQuant-Air-derived and manually scored spike numbers together with root-mean-square error (RMSE), resulting in R^2^ = 0.981 (*P* < 0.001, RMSE = 17.52; **Figure 4B**). The result suggested that the AI-powered spike detection was significantly correlated with the manual scoring, indicating the reliability of the AI-powered detection under field conditions.

Additionally, we compared the spike detection results generated by the optimised YOLOv7 model and seven state-of-the-art object detection models, including YOLOv4, YOLOv5, YOLOX, Faster-RCNN, SSD, RetinaNet, and CenterNet (Zhang et al., 2023), all of which were carefully fine-tuned to yield an optimal wheat spike detection. The detection results produced by the DL models (**Figure 4C**) suggested that the YOLOv7-based model achieved the best accuracy in terms of the mAP@0.5 (i.e. 0.9286), slightly higher than YOLOX (0.9277), YOLOv5 (0.9133), SSD (0.9058), and YOLOv4 (0.9101), demonstrating the biological relevance of applying DL techniques to study spike-like yield components under field conditions.

### The GUI of CropQuant-Air software

**Figure 5:**
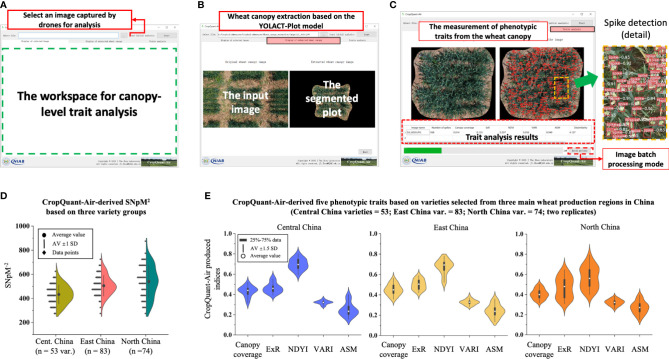
Graphic user interface (GUI) of the CropQuant-Air software system developed for non-expert users together with trait analysis results produced by the system. **(A–C)** The GUI window of the CropQuant-Air system, consisting of input and analysis sections, which could process a single or a series of drone-collected aerial image for plot segmentation and phenotypic analysis, quantifying traits such as SNpM^2^, morphological and spectral traits. **(D, E)** CropQuant-Air-derived traits divided by wheat varieties selected from three main wheat production regions in China, i.e. Central, East and North China.

The CropQuant-Air software system provides a graphical user interface (GUI) that enables non-expert users to perform plot-based trait analysis using an aerial image selected through a unified workspace. A user selects the image selection section (**Figure 5A**) to choose an aerial image. After that, the CropQuant-Air will initiate the display function to visualise the selected image in the workspace for the user to verify the selection. By clicking the ‘Next’ button, the software invokes the plot segmentation module that applied the YOLACT-Plot model to process the selected image, resulting in the central plot in the input image segmented from its surrounding pixels (**Figure 5B**). Depending on the GPU and the size of the selected image, the segmentation process could take up to 10-15 seconds. The final step of the analysis is to carry out wheat spike detection (using the optimised YOLOv7) and canopy-level trait analysis (using the AirMeasurer library), which generates red-coloured binding boxes and confidence levels of all the detected spikes (**Figure 5C**, right), as well as trait analysis results (including SNpM^2^, canopy coverage, ExR, NDYI, VARI, ASM, and GLCM dissimilarity) in a table at the bottom of the workspace (red dotted rectangle; **Figure 5C**, left). The software also supports batch-processing, which can analyse a series of input images and export associated trait analysis (in CSV). Users can download the analysis results and processed images (i.e. segmented plots and detected spikes) via the software. Using an NVIDIA 3060 graphics card, we could achieve a shorter running time (27-30% fasters than CPU-based computation during batch processing) on the CropQuant-Air system compared with an integrated graphics (e.g. Intel’s Iris graphics series) as both plot segmentation and spike detection models were accelerated by GPU through parallel computing.

### Trait analysis using varieties from different production regions

We aimed to apply the CropQuant-Air software to quantify differences between varieties selected from the three wheat production regions (53 varieties from Central China, 83 from East China, and 74 from North China; also see **Supplementary Material**). After processing the 210 varieties, we produced six phenotypic traits relevant to yield components. Comparing the SNpM^2^ trait, while the average value of spike density was slightly different across the three variety groups, increasing between Central (350-500 per m^2^, mean = 410), Eastern (400-550 per m^2^, mean = 500) and Northern wheat varieties (500-575 per m^2^, mean = 530), the distribution for Northern varieties was much more diverse (**Figure 5D**), indicating the large variation of spike density in the variety group.

The same elongation was apparent when comparing performance-related traits, where the spectral traits (i.e. ExR and NDYI) also had a much broader distribution in the selected Northern varieties (**Figure 5E**; right), suggesting varied colour features (i.e. ExG) and developmental paces (i.e. NDYI) among the Northern varieties. The three other measured traits such as canopy coverage, VARI, and ASM were generally similar between the three variety groups according to the violin diagrams. It seemed that the canopy-level phenotypic variation between all the Eastern and Central varieties were relatively small, whereas the Northern varieties possessed much bigger differences (**Figure 5E**). The above observation was applied to the following yield-based analysis and was utilised when building the yield classification model.

### The yield classification in wheat

**Figure 6:**
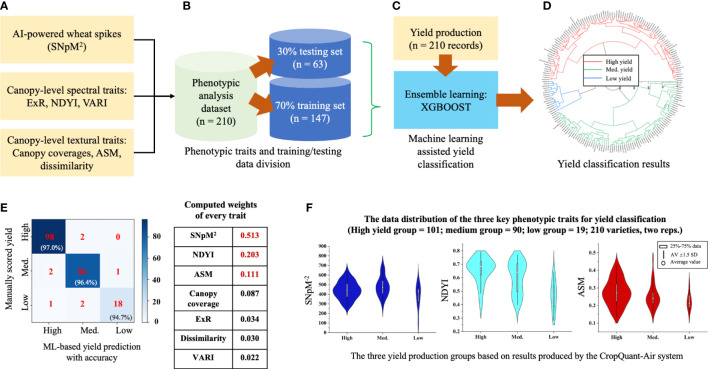
The establishment of the yield classification model and identified key contributing phenotypic traits. **(A–C)** The XGBoost model was used to train the yield classification model with 147 lines and 63 lines for evaluation. **(D)** The model was then applied to classify yield production, resulting in three yield groups. **(E)** Confusion matrix and weights of every trait were computed to verify the classification result and identify key contributing traits. **(F)** Violin diagrams used to represent distributions of the three key traits according to the three yield groups.

To classify wheat yield production for agronomic management reasons (Leilah and Al-Khateeb, 2005), we chose the XGBoost model to perform yield-based classification. We used the trait analysis results (seven parameters; n = 210 records, which was averaged using the two replicates) generated by the CropQuant-Air system as inputs (**Figure 6A**), including SNpM^2^, canopy-level spectral (i.e. ExR, NDYI, VARI) and textural traits (canopy coverage, ASM, dissimilarity). The dataset together with the variety-based yield production data (210 records, derived from plot-based grain production) was then divided into 7:3 ratio, with 70% (147 lines) for training and 30% (63 lines) for testing. When applying the XGBoost learning model to classify yield production, we performed cross-validation in each round of Boosting iteration, enabling the optimal iteration number (**Figures 6B, C**). The yield production was divided into three categories, i.e. high-, medium-, and low-yielding groups, following a published approach for wheat breeding and cultivation (Pantazi et al., 2016).

The trained XGBoost model identified 101 high-yield, 90 medium-yield and 19 low-yield wheat varieties (**Figure 6D**; also see **Table S2** in the **Supplementary Material**). We used confusion matrices to verify the accuracy of the model with manually scored yield production and concluded that: (1) for the high-yielding varieties, the model achieved an accuracy of 97.0%; (2) the medium-yielding group, 96.4% accuracy; (3) the low-yielding group, 94.7% accuracy (**Figure 6E**; left). Moreover, we studied the weights of all the traits in the model and identified that SNpM^2^, NDYI, and ASM jointly contributed 82.7% of the prediction power (coloured red in **Figure 6E**; right), indicating their relevance in wheat yield production. To gain an in-depth understanding of the three traits, we then plotted the distribution of the SNpM^2^, NDYI, and ASM traits using violin diagrams. We could observe that: (1) for the SNpM^2^ trait, spike density of high-yield varieties largely located in the 375-500 region, whereas medium- and low-yield groups had a more diverse spike density; also, low-yield varieties on average had a lower SNpM^2^ value with many low values absent from the other two groups (**Figure 6F**; left); (2) for the NDYI trait, both high- and low-yield varieties had diverse distributions with peaks at 0.68 and 0.45, respectively; the medium-yield varieties followed a double normal distribution with the two peaks corresponding to those in the high- and low-yield groups (**Figure 6F**; middle); (3) for the ASM trait, broadly similar distributions across the 3 yield groups could be observed, with descending means from high to low (**Figure 6F**; right).

## Discussion and conclusion

The ability to identify key phenotypic traits that could be utilised to classify yield production was key for breeders, crop researchers, growers and farmers, and even policymakers as reliable decisions could be rendered to facilitate agronomic management, the selection of crop varieties, and even planning food supply for the market (Chen et al., 2021). For example, understanding the yield potential at key growth stages was essential for breeders to make decisions regarding their crop improvement strategies, helping an efficient identification of genotypes with desired yield- and performance-related traits (Cobb et al., 2013). From cultivation and agronomy’s perspective, yield-based analysis could also lead to the development of more precise agronomic management activities to optimise crop growing conditions and thus improved yield performance (Reynolds et al., 2019). For growers and farmers, to be able to classify yield enabled efficient crop management, providing a baseline to plan agricultural activities (Kremen et al., 2012).

### Standard drone-based phenotyping and AI-powered trait analysis

In our study, we demonstrated that low-cost drones could be utilised to perform standardised aerial imaging to collect canopy-level wheat spikes at key growth stages, whose quality was sufficient for AI-powered plot and spike detection, as well as yield-based classification. To quantify the SNpM^2^ trait, we combined the plot- and spike-level object detection, which was empowered by the AI-based semantic segmentation and vision-based object detection to identify ROIs (i.e. wheat plots) from an aerial image, within which spikes were detected. This progressive algorithmic approach helped us establish an effective workflow to batch-process aerial image series, improving the productivity of the analysis solution presented here. More interestingly, we incorporated spectral and textural features and semantic information of wheat spikes into the model training, which achieved the best detection accuracy compared with seven state-of-the-art DL models, demonstrating a valuable attempt that combined plot- (i.e. the YOLACT-Plot model for instance segmentation) and spike-level (i.e. the optimised YOLOv7 model for semantic segmentation) DL techniques with traditional image processing algorithms to quantify key yield components. To verify the AI-powered trait analysis, we further evaluated the computational results with both manually scored spike number and yield production, resulting in highly significant correlations and thus the reliability of our phenotypic analysis pipeline.

### The open-source platform and yield classification

To enable non-experts to use our solution, we developed an open-source software system called CropQuant-Air, which integrated DL models and image processing algorithms to perform plot-based spike detection, as well as spectral and textural trait analysis with a batch-processing mode. Due to limited toolkits available for nonexperts to examine multigenic traits and develop markers (Sun et al., 2022), we developed the CropQuant-Air system using open scientific libraries, demonstrating the value of open scientific solutions for plant researchers when carrying out phenotypic analysis. The modular design also indicated that all the functions or modules in the CropQuant-Air could be utilised independently, accelerating other academic researchers or developers to build upon our work. Furthermore, we are maintaining the software via our GitHub repository, so that new developments of CropQuant-Air could be promptly shared with the broader plant research community to support other phenotyping research.

To facilitate yield classification in wheat, we produced a separate XGBoost ensemble, through which we identified that the SNpM^2^ trait contributed the most in yield classification and hence the most important factor for yield-related prediction in wheat. Also, subsequent improvements to the ensemble model could include spikelet density, historic yield records, growth stages, and key environmental factors such as ambient temperature and accumulated temperature (Yang et al., 2018), which could improve the generalisation of the yield classification to be applicable during the entire reproductive phase and across different environments.

### Limitation of the study

With the rapid development of multi- and hyper-spectral imaging technologies in recent years, the quality of visible and invisible spectrum imaging has been greatly improved, providing new approaches to image wheat spikes and their development at the canopy level. We used sRGB images to capture wheat spikes’ spectral and morphological features; however, it is likely that multi- and hyper-spectral imagery could obtain more unique spectral signatures of wheat spikes and thus potentially easier to analyse the trait. Additionally, it is worth noting that the RGB images were very limited in detecting plant abiotic or biotic stresses at the spike level, for which hyperspectral sensors could be valuable in studying plant-disease interactions such as the early growth of *Fusarium* within infected wheat spikes (Ninomiya, 2022). Also, our study focused on detecting wheat spikes within breeding plots and did not perform trait analysis under agricultural conditions. Hence, more R&D activities are still required if the CropQuant-Air system needs to be utilised for cultivation and agronomic services.

Another limit of the open scientific platform that could prevent easy-to-access of open scientific work was the Python dependencies. Due to computer vision and DL/ML based software implementation, when sharing, extending, and upgrading our modules in the CropQuant-Air system, it was important to ensure that the correct versions of ML/DL and open scientific libraries were installed. We mitigated the version risk by releasing an executive file (.EXE) of the system, which required us to publish new versions of the executive file if new functions or dependencies were updated. As a result, a community-driven solution might be valuable to develop and improve CropQuant-Air, promoting open and easy-to-use software solutions jointly via the GitHub platform, which could also maximise the impacts of open scientific software R&D in a collaborative manner.

### Future work

Besides the desktop implementation of CropQuant-Air, we could consider deploying the analysis pipeline onto the cloud-based and/or edge computing, so that the software solution could be utilised for different breeding and crop research scenarios. New hardware is also likely to support near real-time analysis based upon our phenotypic analysis solution, providing more economic and powerful tools for agricultural practitioners and researchers. So, key yield-related analysis could be obtained to benchmark yield potential, comparing the performance of different crop varieties and identifying varieties with higher yields under field conditions.

This could also be valuable when identifying crop varieties that were better adapted to local environmental conditions, leading to less water, fertilisers, and other agronomic inputs, which will help growers and farmers reduce the environmental footprint while still maintaining yields. Finally, through big data analytics and cost-effective hardware, much labour-intensive crop surveillance activities could be greatly benefited, facilitating Agri-Food and Agri-Tech companies, policymakers to determine the economic viability of recommended varieties in changing environments, which could also help assess the potential commercial value of the selected varieties so that sound and affordable agricultural production could be promoted.

## Data availability statement

The testing datasets, Jupyter notebook, and CropQuant-Air software used in this paper are available at the Zhou lab’s GitHub repository: https://github.com/The-Zhou-Lab/CropQuant-Air/releases/tag/v1.0. The raw data supporting the conclusions of this article will be made available by the authors, without undue reservation. Other data and user guides are openly available on request.

## Author contributions

JiZ and JC wrote the manuscript with inputs from all the authors; QL, HL, YX, and RJ conducted field experiments and aerial phenotyping under JiZ, GZ, and DJ’s supervision; JiZ, GD, RJ, QL, and JC performed data analysis and statistical analysis; JieZ, JC, GS, and QL built and tested the deep learning models, trait analysis, and the yield classification model under JiZ and DJ’s supervision. JieZ and JC developed the GUI of the CropQuant-Air system with help from GS. JC, JieZ, and QL contributed equally to this work. All authors contributed to the article and approved the submitted version.

## Supplementary material

Supplementary Table 1 The trial design and wheat varieties studied in the 2021/22 season.

Supplementary Table 2 Yield classification classified by the XGBoost model, including 101 high-yielding, 90 medium-yielding, and 19 low-yielding wheat varieties, with manually scored grain production per unit area (GPpM^2^).
