# Supplementary material for: CropQuant-Air: an AI-powered system to enable phenotypic analysis of yield- and performance-related traits using wheat canopy imagery collected by low-cost drones
Source: Front Plant Sci. 2023 Jun 19;14:1219983. doi: 10.3389/fpls.2023.1219983 (PMC10316027; doi:10.3389/fpls.2023.1219983)
Supplement: Supplementary Table 1 — The trial design and wheat varieties studied in the 2021/22 season. [file Table_1.docx]

**Supplementary Material**

**Title: CropQuant-Air: an AI-powered system to enable phenotypic analysis of yield- and performance-related traits using wheat canopy imagery collected by low-cost drones**

In order to assess the performance of the CropQuant-Air system using a real-world dataset, we utilised the varieties below, which are representative lines in main wheat production regions in China. Detailed explanation of these wheat varieties can be found in Betts, A., *et al.* (2014).

**Table S1** The trial design and wheat varieties studied in the 2021/22 season.

| **ID** | **Variety** | **Variety name in Chinese** | **Treatment** | **Production region** |
| --- | --- | --- | --- | --- |
| 1 | Aikang-58 | 矮抗58 | N-240 | Central China |
| 2 | Baomai-10 | 保麦10号 | N-240 | North Chin |
| 3 | Beijing-10 | 北京10号 | N-240 | North Chin |
| 4 | Boai-7023 | 博爱7023 | N-240 | Central China |
| 5 | Chuanmai-22 | 川麦22 | N-240 | Central China |
| 6 | Chuanmai-36 | 川麦36 | N-240 | Central China |
| 7 | Chuanmai-42 | 川麦42 | N-240 | Central China |
| 8 | Dekang-961 | 德抗961 | N-240 | North Chin |
| 9 | Dexuan-1 | 德选1号 | N-240 | North Chin |
| 10 | Emai-11 | 鄂麦11 | N-240 | Central China |
| 11 | Emai-12 | 鄂麦12 | N-240 | Central China |
| 12 | Emai-15 | 鄂麦15 | N-240 | Central China |
| 13 | Emai-19 | 鄂麦19 | N-240 | Central China |
| 14 | Emai-6 | 鄂麦6号 | N-240 | Central China |
| 15 | Fan-6 | 繁6 | N-240 | Central China |
| 16 | Fengdecunmai-1 | 丰德存麦1号 | N-240 | Central China |
| 17 | Fengkang-8 | 丰抗8号 | N-240 | North Chin |
| 18 | Gaoyou-503 | 高优503 | N-240 | North Chin |
| 19 | Guomai-301 | 国麦301 | N-240 | Central China |
| 20 | Han-7086 | 邯7086 | N-240 | North China |
| 21 | Gaoyou-2018 | 蒿优2018 | N-240 | North China |
| 22 | Henong-2063 | 河农2063 | N-240 | North China |
| 23 | Henong-6049 | 河农6049 | N-240 | North China |
| 24 | Henong-825 | 河农825 | N-240 | North China |
| 25 | Heng-136 | 衡136 | N-240 | North China |
| 26 | Heng-4399 | 衡4399 | N-240 | North China |
| 27 | Yangmai-20 | 衡6632 | N-240 | North China |
| 28 | Hengguan-35 | 衡观35 | N-240 | North China |
| 29 | Hengza-102 | 衡杂102 | N-240 | North China |
| 30 | Huaimai-20 | 淮麦20 | N-240 | East China |
| 31 | Huaimai-22 | 淮麦22 | N-240 | East China |
| 32 | Huaimai-28 | 淮麦28 | N-240 | East China |
| 33 | Huaimai-29 | 淮麦29 | N-240 | East China |
| 34 | Huaimai-30 | 淮麦30 | N-240 | East China |
| 35 | Huaimai-33 | 淮麦33 | N-240 | East China |
| 36 | Huaimai-6 | 淮麦6号 | N-240 | East China |
| 37 | Jimai-19 | 济麦19 | N-240 | North China |
| 38 | Jiimai-20 | 济麦20 | N-240 | North China |
| 39 | Jimai-21 | 济麦21 | N-240 | North China |
| 40 | Jimai-22 | 济麦22 | N-240 | North China |
| 41 | Jimai-229 | 济麦229 | N-240 | North China |
| 42 | Jimai-23 | 济麦23 | N-240 | North China |
| 43 | Jinan-16 | 济南16 | N-240 | North China |
| 44 | Jinan-17 | 济南17 | N-240 | North China |
| 45 | Jinan-2 | 济南2号 | N-240 | North China |
| 46 | Jinan-8 | 济南8号 | N-240 | North China |
| 47 | Jinanai-6 | 济南矮6号 | N-240 | North China |
| 48 | Jining-16 | 济宁16号 | N-240 | North China |
| 49 | Hebei-5265 | 冀5265 | N-240 | North China |
| 50 | Hebeimai-19 | 冀麦19 | N-240 | North China |
| 51 | Hebeimai-19 | 冀麦1号 | N-240 | North China |
| 52 | Hebeimai-26 | 冀麦26 | N-240 | North China |
| 53 | Jinhe-9123 | 金禾9123 | N-240 | North China |
| 54 | Jinmai-31 | 晋麦31 | N-240 | North China |
| 55 | Jinmai-33 | 晋麦33 | N-240 | North China |
| 56 | Jimai-47 | 晋麦47 | N-240 | North China |
| 57 | Jingdong-17 | 京冬17 | N-240 | North China |
| 58 | Jingdong-18 | 京冬18 | N-240 | North China |
| 59 | Jingdong-22 | 京冬22 | N-240 | North China |
| 60 | Jinghua-9 | 京花9 | N-240 | North China |
| 61 | Jingshuang-16 | 京双16 | N-240 | North China |
| 62 | Keyuan-008 (Hemai-17) | 科源088（荷麦17号） | N-240 | East China |
| 63 | Laizhou-137 | 莱州137 | N-240 | North China |
| 64 | Laizhou-953 | 莱州953 | N-240 | North China |
| 65 | Lianmai-2 | 连麦2号 | N-240 | East China |
| 66 | Lianmai-6 | 连麦6号 | N-240 | East China |
| 67 | Lianmai-7 | 连麦7号 | N-240 | East China |
| 68 | Liangxing-66 | 良星66 | N-240 | North China |
| 69 | Liangxing-77 | 良星77 | N-240 | North China |
| 70 | Lin-Y8012 (Pinyu-8012) | 临Y8012(**品育8012)** | N-240 | North China |
| 71 | Linmai-4 | 临麦4 | N-240 | North China |
| 72 | Lukenmai-9 | 鲁垦麦9 | N-240 | North China |
| 73 | Lumai-14 | 鲁麦14 | N-240 | North China |
| 74 | Lumai-15 | 鲁麦15 | N-240 | North China |
| 75 | Lumai-1 | 鲁麦1号 | N-240 | North China |
| 76 | Lumai-21 | 鲁麦21 | N-240 | North China |
| 77 | Lumai-22 | 鲁麦22 | N-240 | North China |
| 78 | Lumai-5 | 鲁麦5号 | N-240 | North China |
| 79 | Luyuan-502 | 鲁原502 | N-240 | North China |
| 80 | Lunxuan-987 | 轮选987 | N-240 | North China |
| 81 | Luohan-11 | 洛旱11号 | N-240 | North China |
| 82 | Luohan-13 | 洛旱13号 | N-240 | North China |
| 83 | Luohan-2 | 洛旱2 | N-240 | North China |
| 84 | Luohan-9 | 洛旱9号 | N-240 | North China |
| 85 | Luomai-21 | 洛麦21号 | N-240 | North China |
| 86 | Luomai-26 | 洛麦26 | N-240 | North China |
| 87 | Luomai-7 | 洛麦7号（漯麦7号） | N-240 | North China |
| 88 | Machang-2 | 马场2号 | N-240 | East China |
| 89 | Mianmai-37 | 绵麦37 | N-240 | Central China |
| 90 | Mianmai-39 | 绵麦39 | N-240 | Central China |
| 91 | Xumai-32 | 绵阳11 | N-240 | Central China |
| 92 | Nannong-0686 | 南农0686 | N-240 | East China |
| 93 | Ningmai-13 | 宁麦13 | N-240 | East China |
| 94 | Ningmai-15 | 宁麦15 | N-240 | East China |
| 95 | Ningmai-22 | 宁麦22 | N-240 | East China |
| 96 | Ningmai-24 | 宁麦24 | N-240 | East China |
| 97 | Ningmai-26 | 宁麦26 | N-240 | East China |
| 98 | Ningmai-9 | 宁麦9号 | N-240 | East China |
| 99 | Ningrumai-1 | 宁糯麦1号 | N-240 | East China |
| 100 | Nongda-211 | 农大211 | N-240 | Central China |
| 101 | Qida-195 | 齐大195 | N-240 | North China |
| 102 | Qimai-2 | 齐麦2号 | N-240 | North China |
| 103 | Qingnong-2 | 青农2号 | N-240 | North China |
| 104 | Rumai-1 | 儒麦1号 | N-240 | North China |
| 105 | Ruihuamai-520 | 瑞华麦520 | N-240 | East China |
| 106 | Ruihuamai-523 | 瑞华麦523 | N-240 | East China |
| 107 | Shannong-12 | 山农12号 | N-240 | North China |
| 108 | Shannong-15 | 山农15 | N-240 | North China |
| 109 | Shannong-20 | 山农20 | N-240 | North China |
| 110 | Shannong-205 | 山农205 | N-240 | North China |
| 111 | Shannong-21 | 山农21 | N-240 | North China |
| 112 | Shannong-22 | 山农22 | N-240 | North China |
| 113 | Shannong-24 | 山农24 | N-240 | North China |
| 114 | Shannong-28 | 山农28 | N-240 | North China |
| 115 | Shannong-29 | 山农29 | N-240 | North China |
| 116 | Shengxuan-3 | 生选3号 | N-240 | East China |
| 117 | Shengxuan-6 | 生选6号 | N-240 | East China |
| 118 | Shiluan-02-1 | 师栾02-1 | N-240 | North China |
| 119 | Shi-10-4393-14 (Shimai-26) | 石10-4393-14（石麦26） | N-240 | North China |
| 120 | Shi-12-4025 (Shimai-28) | 石12-4025（石麦28） | N-240 | North China |
| 121 | Shi-4185 | 石4185 | N-240 | North China |
| 122 | Shijiazhuang-407 | 石家庄407 | N-240 | North China |
| 123 | Shijiazhuang-8 | 石家庄8号 | N-240 | North China |
| 124 | Shimai-12 | 石麦12 | N-240 | North China |
| 125 | Shimai-15 | 石麦15 | N-240 | North China |
| 126 | Shimai-18 | 石麦18 | N-240 | North China |
| 127 | Shimai-22 | 石麦22 | N-240 | North China |
| 128 | Shiyou-20 | 石优20 | N-240 | North China |
| 129 | Shunmai-1718 | 舜麦1718 | N-240 | North China |
| 130 | Sumai-188 | 苏麦188 | N-240 | East China |
| 131 | Sumai-3 | 苏麦3号 | N-240 | East China |
| 132 | Tai-10604 | 太10604 | N-240 | North China |
| 133 | Taikemai-31 | 泰科麦31 | N-240 | North China |
| 134 | Taikemai-33 | 泰科麦33 | N-240 | North China |
| 135 | Taimai-1918 (Taimai-198) | 泰麦1918（太麦198） | N-240 | North China |
| 136 | Tainong-19 | 泰农19 | N-240 | North China |
| 137 | Taishan-1 | 泰山1号 | N-240 | North China |
| 138 | Taishan-27 | 泰山27 | N-240 | North China |
| 139 | Taishan-28 | 泰山28 | N-240 | North China |
| 140 | Taishan-4 | 泰山4号 | N-240 | North China |
| 141 | Taishan-5366 | 泰山5366 | N-240 | North China |
| 142 | Taitianmai-118 | 泰田麦118 | N-240 | North China |
| 143 | Tainong-24 | 郯农98 | N-240 | North China |
| 144 | Wanmai-0066 | 皖麦0066 | N-240 | East China |
| 145 | Wanmai-19 | 皖麦19 | N-240 | East China |
| 146 | Wanmai-33 | 皖麦33 | N-240 | East China |
| 147 | Wanmai-50 | 皖麦50 | N-240 | East China |
| 148 | Wanximai-0638 | 皖西麦0638 | N-240 | East China |
| 149 | Wanfeng-269 | 万丰269 | N-240 | Central China |
| 150 | Wannian-2 | 万年2号 | N-240 | Central China |
| 151 | Wennong-14 | 汶农14 | N-240 | North China |
| 152 | Wennong-5 | 汶农5 | N-240 | North China |
| 153 | Wennong-17 | 汶农17 | N-240 | North China |
| 154 | Xinmai-16 | 新麦16 | N-240 | North China |
| 155 | Xinmai-18 | 新麦18 | N-240 | North China |
| 156 | Xinmai-20 | 新麦20 | N-240 | North China |
| 157 | Xinmai-9 | 新麦9号 | N-240 | North China |
| 158 | Xinmai  (sanjin)-296 | 鑫麦296 | N-240 | North China |
| 159 | Xingmai-18 | 邢麦18 | N-240 | North China |
| 160 | Xingmai-7 | 邢麦7号 | N-240 | North China |
| 161 | Xumai-30 | 徐麦30 | N-240 | East China |
| 162 | Xumai-33 | 徐麦33 | N-240 | East China |
| 163 | Xuzhou-24 | 徐州24 | N-240 | East China |
| 164 | Xuzhou-438 | 徐州438 | N-240 | East China |
| 165 | Xuzhou-8 | 徐州8号 | N-240 | East China |
| 166 | Yanfu-188 | 烟辐188 | N-240 | North China |
| 167 | Yannong-0428 | 烟农0428 | N-240 | North China |
| 168 | Yannong-1212 | 烟农1212 | N-240 | North China |
| 169 | Yannong-15 | 烟农15 | N-240 | North China |
| 170 | Yannong-173 | 烟农173 | N-240 | North China |
| 171 | Yannong-19 | 烟农19 | N-240 | North China |
| 172 | Yannong-22 | 烟农22号 | N-240 | North China |
| 173 | Yannong-836 | 烟农836 | N-240 | North China |
| 174 | Yannong-999 | 烟农999 | N-240 | North China |
| 175 | Yangmai-10 | 扬麦10号 | N-240 | East China |
| 176 | Yangmai-13 | 扬麦13 | N-240 | East China |
| 177 | Yangmai-15 | 扬麦15 | N-240 | East China |
| 178 | Yangmai-158 | 扬麦158 | N-240 | East China |
| 179 | Yangmai-16 | 扬麦16 | N-240 | East China |
| 180 | Yangmai-18 | 扬麦18 | N-240 | East China |
| 181 | Yangmai-1 | 扬麦1号 | N-240 | East China |
| 182 | Yangmai-22 | 扬麦22 | N-240 | East China |
| 183 | Yangmai-5 | 扬麦5号 | N-240 | East China |
| 184 | Yangmai-9 | 扬麦9号 | N-240 | East China |
| 185 | Yumai-13 | 豫麦13 | N-240 | Central China |
| 186 | Yumai-18 | 豫麦18 | N-240 | Central China |
| 187 | Yumai-2 | 豫麦2号 | N-240 | Central China |
| 188 | Yunhan-2233 | 运旱2233 | N-240 | North China |
| 189 | Yunhan-618 | 运旱618 | N-240 | North China |
| 190 | Xumai-31 | 长4640 | N-240 | North China |
| 191 | Chang-4738 | 长4738 | N-240 | North China |
| 192 | Zhemai-1 | 浙麦1号 | N-240 | East China |
| 193 | Zhenmai-12 | 镇麦12号 | N-240 | East China |
| 194 | Zhenmai-168 | 镇麦168 | N-240 | East China |
| 195 | Zhenmai-3 | 镇麦3号 | N-240 | East China |
| 196 | Zhenmai-4 | 镇麦4号 | N-240 | East China |
| 197 | Zhenmai-9 | 镇麦9号 | N-240 | East China |
| 198 | Zhengmai-004 | 郑麦004 | N-240 | Central China |
| 199 | Zhengmai-7698 | 郑麦7698 | N-240 | Central China |
| 200 | Zhengmai-9023 | 郑麦9023 | N-240 | Central China |
| 201 | Zhongmai-175 | 中麦175 | N-240 | North China |
| 202 | Zhongmai-9 | 中麦9号 | N-240 | North China |
| 203 | Zhoumai-12 | 周麦12 | N-240 | Central China |
| 204 | Zhoumai-18 | 周麦18 | N-240 | Central China |
| 205 | Zhoumai-23 | 周麦23 | N-240 | Central China |
| 206 | Zhoumai-24 | 周麦24 | N-240 | Central China |
| 207 | Zhoumai-26 | 周麦26 | N-240 | Central China |
| 208 | Zhoumai-27 | 周麦27 | N-240 | Central China |
| 209 | Zhoumai-32 | 周麦32 | N-240 | Central China |
| 210 | Zinong-033 | 淄农033 | N-240 | North China |

**Table S2** Yield classification classified by the XGBoost model, including 101 high-yielding, 90 medium-yielding, and 19 low-yielding wheat varieties, with manually scored grain production per unit area (GPpM^2^).

| **High-yielding group (101 varieties)** | | | | | |
| --- | --- | --- | --- | --- | --- |
| **Serial number** | **Variety Name** | **Yield**  **(GPpM^2^)** | **Serial number** | **Variety Name** | **Yield**  **(GPpM^2^)** |
| 1 | Shi-4185 | 1.3855 | 52 | Yannong-22 | 1.412 |
| 2 | Shijiazhuang-407 | 1.2295 | 53 | Yannong-836 | 1.427 |
| 3 | Shijiazhuang-8 | 0.9675 | 54 | Yannong-999 | 1.496 |
| 4 | Shimai-12 | 1.1255 | 55 | Yangmai-10 | 1.612 |
| 5 | Shimai-15 | 1.1185 | 56 | Yangmai-13 | 1.4625 |
| 6 | Shimai-18 | 1.18 | 57 | Yangmai-15 | 1.597 |
| 7 | Shimai-22 | 1.1425 | 58 | Yangmai-158 | 1.2805 |
| 8 | Shiyou-20 | 1.0205 | 59 | Yangmai-16 | 1.63 |
| 9 | Shunmai-1718 | 1.182 | 60 | Yangmai-18 | 1.5785 |
| 10 | Sumai-188 | 1.247 | 61 | Yangmai-1 | 1.364 |
| 11 | Sumai-3 | 1.0205 | 62 | Yangmai-22 | 1.4435 |
| 12 | Tai-10604 | 1.0725 | 63 | Yangmai-5 | 1.2365 |
| 13 | Taikemai-31 | 1.3125 | 64 | Yangmai-9 | 1.281 |
| 14 | Taikemai-33 | 0.861 | 65 | Yumai-13 | 1.02 |
| 15 | Taimai-1918 (Taimai-198) | 1.5525 | 66 | Yumai-18 | 1.189 |
| 16 | Tainong-19 | 1.116 | 67 | Yumai-2 | 0.9095 |
| 17 | Taishan-1 | 1.3165 | 68 | Yunhan-2233 | 1.1635 |
| 18 | Taishan-27 | 1.025 | 69 | Yunhan-618 | 1.3715 |
| 19 | Taishan-28 | 1.106 | 70 | Xumai-31 | 0.9995 |
| 20 | Taishan-4 | 1.2005 | 71 | Chang-4738 | 1.2655 |
| 21 | Taishan-5366 | 1.272 | 72 | Zhemai-1 | 1.674 |
| 22 | Taitianmai-118 | 1.369 | 73 | Zhenmai-12 | 1.472 |
| 23 | Tainong-24 | 1.4575 | 74 | Zhenmai-168 | 1.3985 |
| 24 | Wanmai-0066 | 1.2235 | 75 | Zhenmai-3 | 1.3115 |
| 25 | Wanmai-19 | 1.148 | 76 | Zhenmai-4 | 1.114 |
| 26 | Wanmai-33 | 1.2885 | 77 | Zhenmai-9 | 1.356 |
| 27 | Wanmai-50 | 1.2245 | 78 | Zhengmai-004 | 1.1505 |
| 28 | Wanximai-0638 | 1.228 | 79 | Zhengmai-7698 | 1.1645 |
| 29 | Wanfeng-269 | 0.9865 | 80 | Zhengmai-9023 | 1.0735 |
| 30 | Wannian-2 | 1.2135 | 81 | Zhongmai-175 | 1.087 |
| 31 | Wennong-14 | 0.9535 | 82 | Machang-2 | 1.5285 |
| 32 | Wennong-5 | 0.971 | 83 | Mianmai-37 | 1.4975 |
| 33 | Wennong-17 | 1.2385 | 84 | Mianmai-39 | 1.331 |
| 34 | Xinmai-16 | 1.307 | 85 | Xumai-32 | 1.467 |
| 35 | Xinmai-18 | 0.717 | 86 | Nannong-0686 | 1.3945 |
| 36 | Xinmai-20 | 0.689 | 87 | Ningmai-13 | 1.5355 |
| 37 | Xinmai-9 | 0.76 | 88 | Ningmai-15 | 1.449 |
| 38 | Xinmai (sanjin)-296 | 0.9575 | 89 | Ningmai-22 | 1.708 |
| 39 | Xingmai-18 | 0.926 | 90 | Ningmai-24 | 1.5515 |
| 40 | Xingmai-7 | 1.1725 | 91 | Ningmai-26 | 1.667 |
| 41 | Xumai-30 | 1.4185 | 92 | Ningmai-9 | 1.4695 |
| 42 | Xumai-33 | 1.691 | 93 | Ningrumai-1 | 1.4745 |
| 43 | Xuzhou-24 | 1.577 | 94 | Nongda-211 | 1.3885 |
| 44 | Xuzhou-438 | 0.9225 | 95 | Qida-195 | 1.1535 |
| 45 | Xuzhou-8 | 1.291 | 96 | Qimai-2 | 1.4315 |
| 46 | Yanfu-188 | 1.189 | 97 | Zhoumai-18 | 0.907 |
| 47 | Yannong-0428 | 1.516 | 98 | Zhoumai-23 | 1.1545 |
| 48 | Yannong-1212 | 1.175 | 99 | Zhoumai-24 | 0.767 |
| 49 | Yannong-15 | 1.147 | 100 | Zhoumai-26 | 1.2135 |
| 50 | Yannong-173 | 1.1005 | 101 | Zhoumai-27 | 1.2635 |
| 51 | Yannong-19 | 1.4495 |  |  |  |

| **Medium-yielding group (90 varieties)** | | | | | |
| --- | --- | --- | --- | --- | --- |
| **Serial number** | **Variety Name** | **Yield**  **(GPpM^2^)** | **Serial number** | **Variety Name** | **Yield**  **(GPpM^2^)** |
| 1 | Aikang-58 | 0.9725 | 46 | Lianmai-2 | 0.9255 |
| 2 | Baomai-10 | 1.0225 | 47 | Lianmai-6 | 1.06 |
| 3 | Beijing-10 | 1.0615 | 48 | Lianmai-7 | 0.776 |
| 4 | Boai-7023 | 0.983 | 49 | Liangxing-66 | 0.693 |
| 5 | Chuanmai-22 | 1.1775 | 50 | Liangxing-77 | 0.6625 |
| 6 | Chuanmai-36 | 1.054 | 51 | Lin-Y8012 (Pinyu-8012) | 0.338 |
| 7 | Chuanmai-42 | 1.0325 | 52 | Linmai-4 | 0.868 |
| 8 | Dekang-961 | 0.6075 | 53 | Lukenmai-9 | 0.814 |
| 9 | Dexuan-1 | 0.7315 | 54 | Lumai-14 | 0.106 |
| 10 | Emai-11 | 0.686 | 55 | Lumai-15 | 0.7825 |
| 11 | Emai-12 | 1.306 | 56 | Lumai-1 | 0.7365 |
| 12 | Emai-15 | 0.9235 | 57 | Lumai-21 | 0.9805 |
| 13 | Emai-19 | 0.9235 | 58 | Lumai-22 | 0.7685 |
| 14 | Huaimai-29 | 1.117 | 59 | Lumai-5 | 0.7595 |
| 15 | Huaimai-30 | 0.8645 | 60 | Luyuan-502 | 1.1985 |
| 16 | Huaimai-33 | 0.909 | 61 | Lunxuan-987 | 1.206 |
| 17 | Huaimai-6 | 0.996 | 62 | Luohan-11 | 1.1795 |
| 18 | Jimai-19 | 0.588 | 63 | Luohan-13 | 0.9895 |
| 19 | Jiimai-20 | 1.2085 | 64 | Luohan-2 | 1.145 |
| 20 | Jimai-21 | 1.1065 | 65 | Luohan-9 | 1.265 |
| 21 | Jimai-22 | 0.907 | 66 | Luomai-21 | 1.2655 |
| 22 | Jimai-229 | 0.995 | 67 | Luomai-26 | 1.241 |
| 23 | Jimai-23 | 1.022 | 68 | Luomai-7 | 1.2485 |
| 24 | Jinan-16 | 1.017 | 69 | Qingnong-2 | 1.3595 |
| 25 | Jinan-17 | 0.9465 | 70 | Rumai-1 | 1.2325 |
| 26 | Jinan-2 | 0.8195 | 71 | Ruihuamai-520 | 1.1925 |
| 27 | Jinan-8 | 1.0105 | 72 | Ruihuamai-523 | 1.6365 |
| 28 | Jinanai-6 | 0.9995 | 73 | Shannong-12 | 0.796 |
| 29 | Jining-16 | 0.9075 | 74 | Shannong-15 | 1.3055 |
| 30 | Hebei-5265 | 1.3045 | 75 | Shannong-20 | 1.264 |
| 31 | Hebeimai-19 | 0.953 | 76 | Shannong-205 | 1.238 |
| 32 | Hebeimai-19 | 0.807 | 77 | Shannong-21 | 0.945 |
| 33 | Hebeimai-26 | 0.889 | 78 | Shannong-22 | 1.21 |
| 34 | Jinhe-9123 | 1.1075 | 79 | Shannong-24 | 1.337 |
| 35 | Jinmai-31 | 1.0985 | 80 | Shannong-28 | 1.005 |
| 36 | Jinmai-33 | 1.2065 | 81 | Shannong-29 | 1.2545 |
| 37 | Jimai-47 | 0.9255 | 82 | Shengxuan-3 | 1.4075 |
| 38 | Jingdong-17 | 1.0045 | 83 | Shengxuan-6 | 1.3555 |
| 39 | Jingdong-18 | 1.1065 | 84 | Shiluan-02-1 | 1.2275 |
| 40 | Jingdong-22 | 0.979 | 85 | Shi-10-4393-14 (Shimai-26) | 1.113 |
| 41 | Jinghua-9 | 0.821 | 86 | Shi-12-4025 (Shimai-28) | 1.3465 |
| 42 | Jingshuang-16 | 0.801 | 87 | Zhongmai-9 | 0.9155 |
| 43 | Keyuan-008 (Hemai-17) | 0.585 | 88 | Zhoumai-12 | 0.765 |
| 44 | Laizhou-137 | 0.94 | 89 | Zhoumai-32 | 0.8415 |
| 45 | Laizhou-953 | 0.9915 | 90 | Zinong-033 | 0.9565 |

| **Low-yielding group (19 varieties)** | | | | | |
| --- | --- | --- | --- | --- | --- |
| **Serial number** | **Variety Name** | **Yield**  **(GPpM^2^)** | **Serial number** | **Variety Name** | **Yield**  **(GPpM^2^)** |
| 1 | Emai-6 | 0.942 | 11 | Henong-825 | 0.961 |
| 2 | Fan-6 | 1.087 | 12 | Heng-136 | 0.77 |
| 3 | Fengdecunmai-1 | 1.0825 | 13 | Heng-4399 | 1.0755 |
| 4 | Fengkang-8 | 1.185 | 14 | Yangmai-20 | 0.8115 |
| 5 | Gaoyou-503 | 1.0175 | 15 | Hengguan-35 | 1.157 |
| 6 | Guomai-301 | 1.104 | 16 | Hengza-102 | 0.659 |
| 7 | Han-7086 | 0.881 | 17 | Huaimai-20 | 1.0235 |
| 8 | Gaoyou-2018 | 1.0335 | 18 | Huaimai-22 | 1.053 |
| 9 | Henong-2063 | 0.913 | 19 | Huaimai-28 | 1.078 |
| 10 | Henong-6049 | 0.7915 |  |  |  |

**Reference**

Betts, A., Jia, P. W., and Dodson, J. (2014). The origins of wheat in China and potential pathways for its introduction: A review. *Quat. Int.* 348, 158–168. doi: 10.1016/j.quaint.2013.07.044.
